# Replication of Dengue Virus in K562-Megakaryocytes Induces Suppression in the Accumulation of Reactive Oxygen Species

**DOI:** 10.3389/fmicb.2021.784070

**Published:** 2022-01-11

**Authors:** Jaskaran Kaur, Yogita Rawat, Vikas Sood, Neha Periwal, Deepak Kumar Rathore, Shrikant Kumar, Niraj Kumar, Sankar Bhattacharyya

**Affiliations:** ^1^Translational Health Science and Technology Institute, National Capital Region (NCR) Biotech Science Cluster, Faridabad, India; ^2^Department of Biochemistry, School of Chemical and Life Sciences, Jamia Hamdard (Hamdard University), New Delhi, India

**Keywords:** Dengue virus replication, megakaryopoiesis, reactive oxygen species, NFE2L2, flavivirus

## Abstract

Dengue virus can infect human megakaryocytes leading to decreased platelet biogenesis. In this article, we report a study of Dengue replication in human K562 cells undergoing PMA-induced differentiation into megakaryocytes. PMA-induced differentiation in these cells recapitulates steps of megakaryopoiesis including gene activation, expression of CD41/61 and CD61 platelet surface markers and accumulation of intracellular reactive oxygen species (ROS). Our results show differentiating megakaryocyte cells to support higher viral replication without any apparent increase in virus entry. Further, Dengue replication suppresses the accumulation of ROS in differentiating cells, probably by only augmenting the activity of the transcription factor NFE2L2 without influencing the expression of the coding gene. Interestingly pharmacological modulation of NFE2L2 activity showed a simultaneous but opposite effect on intracellular ROS and virus replication suggesting the former to have an inhibitory effect on the later. Also cells that differentiated while supporting intracellular virus replication showed reduced level of surface markers compared to uninfected differentiated cells.

## Introduction

Dengue virus (DENV) infection causes a self-limiting acute febrile illness with the potential to turn fatal. Bone marrow (BM) megakaryocytes are known to be highly permissible to DENV replication and myelosuppression and a reduction in the mass of BM cells is one of the hallmarks of DENV infection (Clark et al., 2012; Noisakran et al., 2012; Sridharan et al., 2013; Vogt et al., 2019). Independent of disease severity, DENV infection causes thrombocytopenia to a lesser or greater extent with severe patients exhibiting acute drop in platelet level along with leakage of fluid from the blood vessels (Martina et al., 2009). Multiple mechanisms have been suggested to contribute to thrombocytopenia including attenuated platelet biogenesis and increased platelet decay through either direct virus interaction or upon binding of anti-platelet antibodies (Fang et al., 2013; de Azeredo et al., 2015; Simon et al., 2015; Ojha et al., 2017, 2019). Platelets in the blood are produced from specialized bone-marrow resident cells called Megakaryocytes (MKs), at a steady rate of biogenesis (about 1 × 10^9^/day) and with an average life of 7 days in humans (Patel et al., 2005). MKs are generated from bi-potential Megakaryocyte-Erythrocyte progenitor (MEP) cells in bone marrow, by profound cellular and molecular changes including expansion, endomitosis which generates a multi-lobed and polyploid nucleus and expression of platelet specific surface markers on the plasma membrane which become part of the platelets' membrane (Schulze and Shivdasani, 2004; Deutsch and Tomer, 2006). In addition to plasma membrane, platelets bud off from the MK along with a bit of its cytoplasm, containing granules of different types and a host of specific mRNAs (Cecchetti et al., 2011). Multiple transcription factors (TFs) including GATA-1 and RUNX1 also play a crucial role in megakaryopoiesis (Muntean et al., 2007; Lordier et al., 2012). In addition to this, cellular pathways like ER-stress and Autophagy have been shown to be involved in MK differentiation (Lopez et al., 2013).

During megakaryopoiesis, reactive oxygen species (ROS) acts as an important signaling molecule (Chen et al., 2013). In fact, increased oxygen level and augmented expression of ROS generating enzymes show positive correlation with megakaryocyte development, corroborating ROS to serve as a critical promoter of megakaryopoiesis (Mostafa et al., 2000; McCrann et al., 2009; Sardina et al., 2010). ROS has been suggested to function at multiple levels, which include increasing the level of tyrosine-phosphorylation on specific proteins, inhibition of certain tyrosine phosphatases, ERK activation, modulation of cyclin levels and TFs and promoting differentiation associated apoptosis (Sattler et al., 1999; Eliades et al., 2010; Sardina et al., 2010; Siddiqui et al., 2011). Increase in intracellular ROS activates the expression of genes that code for anti-oxidant proteins through the mediation of the transcription factor (TF) Nuclear factor-erythroid factor 2-related factor 2 (NFE2L2) or Nrf2 (Hayes and Dinkova-Kostova, 2014). NFE2L2 can potentially be regulated by transcriptional, post-transcriptional or post-translational means (Zang et al., 2020). In the post-translational mechanism, accumulation of ROS triggers activation of cellular antioxidant genes by disrupting the cytosolic complex between the TF, NFE2L2 and its suppressor KEAP1. Oxidation of KEAP1 releases NFE2L2 from the complex, which migrates to the nucleus to activate many canonical antioxidant genes (Chen et al., 2007). NFE2L2 has been found to be important in the development of MKs as well (Gasiorek and Blank, 2015). However, the level of NFE2L2 target genes are suppressed during later stages of MK differentiation (Chen et al., 2007).

K562 cells represent a bi-potential cell line which can be differentiated to either MK-like cells or Erythroblast-like cells, depending on differential pharmacological stimulation (Witt et al., 2000). Supplementation of Phorbol esters like Phorbol-12 myristate-13 (PMA) in growth media drives differentiation of K562 cells toward MKs, inducing cellular and molecular changes akin to MKs *in vivo*, through activation of identical signaling axes (Lee et al., 1999; Rojnuckarin et al., 1999; Kim et al., 2001; Colosetti et al., 2009; Limb et al., 2009; Ojima et al., 2013; Huang et al., 2014; Nurhayati et al., 2014; Chaman et al., 2015). In addition to this, these cells are permissible to DENV replication and therefore, considered as a good model system to study the effect of DENV infection on megakaryopoiesis (Lin et al., 1998). Previous reports have suggested megakaryocytes to be highly permissive to Dengue replication and infection of megakaryocytes might be responsible for attenuating platelet biogenesis. However, the molecular mechanisms of such virus induced effect is still not clear. In this report we show that DENV replicates better in megakaryocytes and virus infection interferes with accumulation of ROS, thereby potentially inhibiting platelet biogenesis.

## Results

### K562 Cells Recapitulate Major Events in Megakaryopoiesis Upon PMA-Treatment

**Figure 1:**
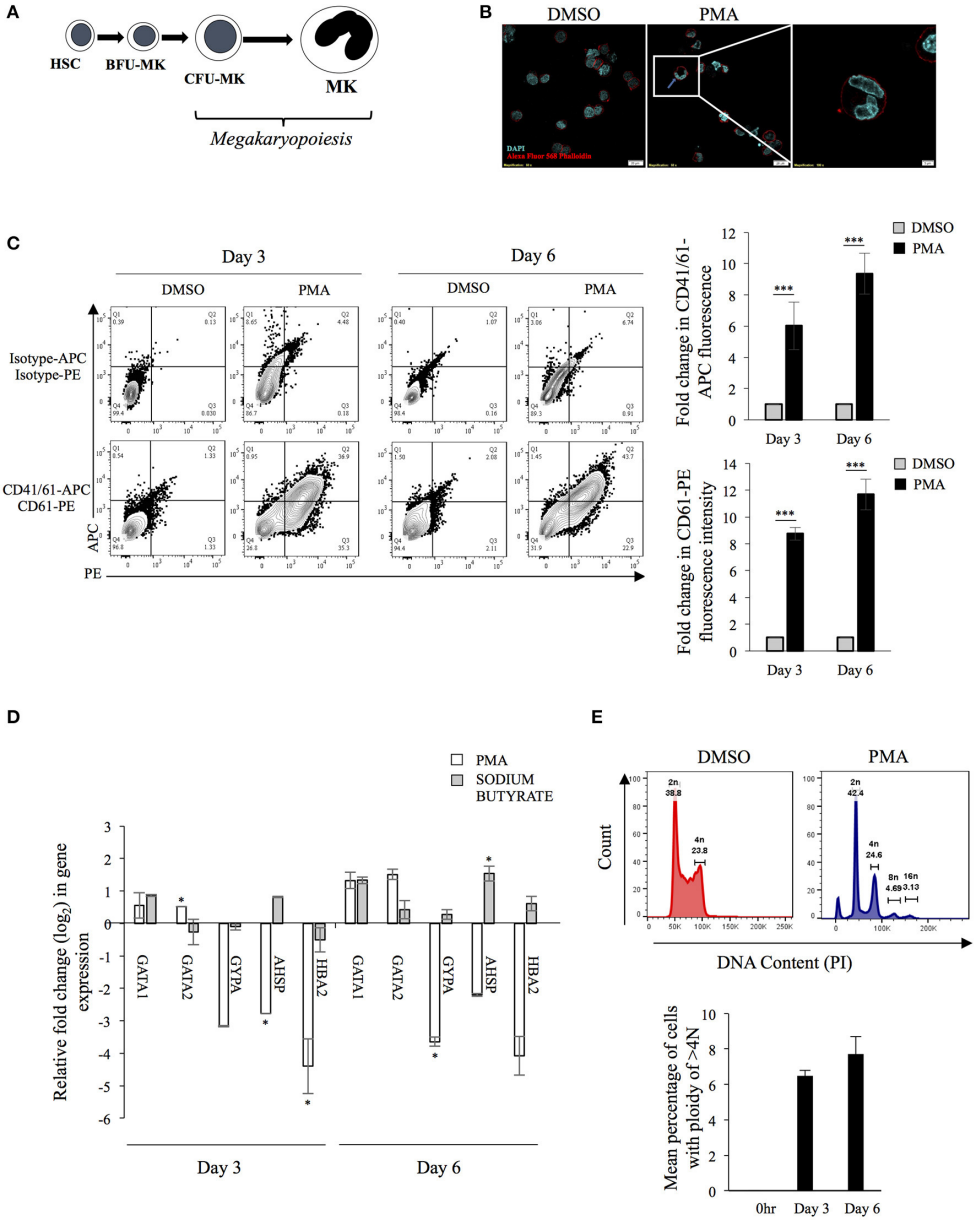
**(A)** Schematic diagram of megakaryopoiesis: The schematic diagram represents select stages during the differentiation of Hematopoietic stem cells (HSC) to Megakaryocytes (MKs) through Blast-forming unit-Mk (BFU-MK) and Colony-forming unit-Mk (CFU-MK). Cell enlargement and formation of multilobed nucleus has been depicted. **(B)** Formation of multi-lobed nucleus following PMA induced differentiation of K562 cells. K562 cells treated with either DMSO or 50 nM PMA for 6 days were stained with Alexa Fluor-568 Phalloidin and DAPI and visualized in a confocal microscope. The red color indicates the plasma membrane and blue color shows nuclei. The inset shows a cell with multilobed nucleus. **(C)** Expression of platelet specific surface markers: K562 cells treated with PMA for 0, 3, or 6 days were immune-stained for expression of the indicated surface marker protein using fluor-conjugated primary antibodies. The mean fluorescence intensity (MFI) corresponding to each day was quantified. For each surface marker the MFI at day 0 was arbitrarily set to 1 and those at day 3 and 6, expressed as the relative fold change with respect to that. **(D)** Total RNA from K562 cells treated with either DMSO or 50 nM PMA or 1 mM sodium butyrate for 3 or 6 days was extracted and purified. The RNA was reverse transcribed with random hexamers and the cDNA used for real-time PCR estimation of mRNA transcripts from indicated genes. The C_T_ value corresponding to each was normalized to that of GAPDH. The normalized value at day 0 was taken arbitrarily as 1 and those at day 3 and 6 expressed as fold-change with respect to that. For Panels A to E, the error bars represent standard deviation obtained from 3 independent experiments. The significance was calculated by Student's *t*-test (*, ***, respectively, indicate *P*-values <0.05 and <0.001). **(E)** K562 cells treated with PMA for 0 or 3 or 6 days were fixed, permeabilized and stained for intracellular DNA using Propidium iodide (PI). The PI stain was quantified by flow cytometry and mean number of cells having ploidy of >4N were plotted.

The differentiation of MKs involves changes in cell size, gene expression and endomitosis as depicted in Figure 1A. Human K562 cells can be induced to differentiate into either megakaryocytes (using phorbol esters) or erythrocytes lineage (using sodium butyrate) (Witt et al., 2000). Upon Phorbol-12 Myristate-13 Acetate (PMA) supplementation cells stopped proliferating, increased attachment to substratum (data not shown), enlarged in size and underwent endomitosis (Figure 1B). The surface expression of two platelet specific markers namely, CD41/61 heterodimer and CD61 showed significant increase (Figure 1C). Real-time PCR based investigation, into the differential expression of genes selected from a study of published literature, showed the expression of certain unique MK-specific genes to be upregulated by PMA but not by sodium butyrate (Li et al., 2008). As expected, transcripts from the transcription factor (TF) gene GATA1 showed upregulation with both drugs, whereas those from GATA2 and CD61 showed increase with PMA but remained unchanged or was suppressed with sodium butyrate (Figure 1D) (Mouthon et al., 1993; Ikonomi et al., 2000). Similarly, expression of GYPA, HBA2 and AHSP increased with sodium butyrate but decreased with PMA (Kawasaki et al., 1996) (Figure 1D). Analysis of the cellular ploidy level showed the emergence of polyploid cells with 8N and 16N (Figure 1E). These results corroborated that K562 cells are of bi-potential nature and upon PMA treatment differentiate into MKs.

### Differentiation of K562-MKs Promote Replication of DENV

**Figure 2:**
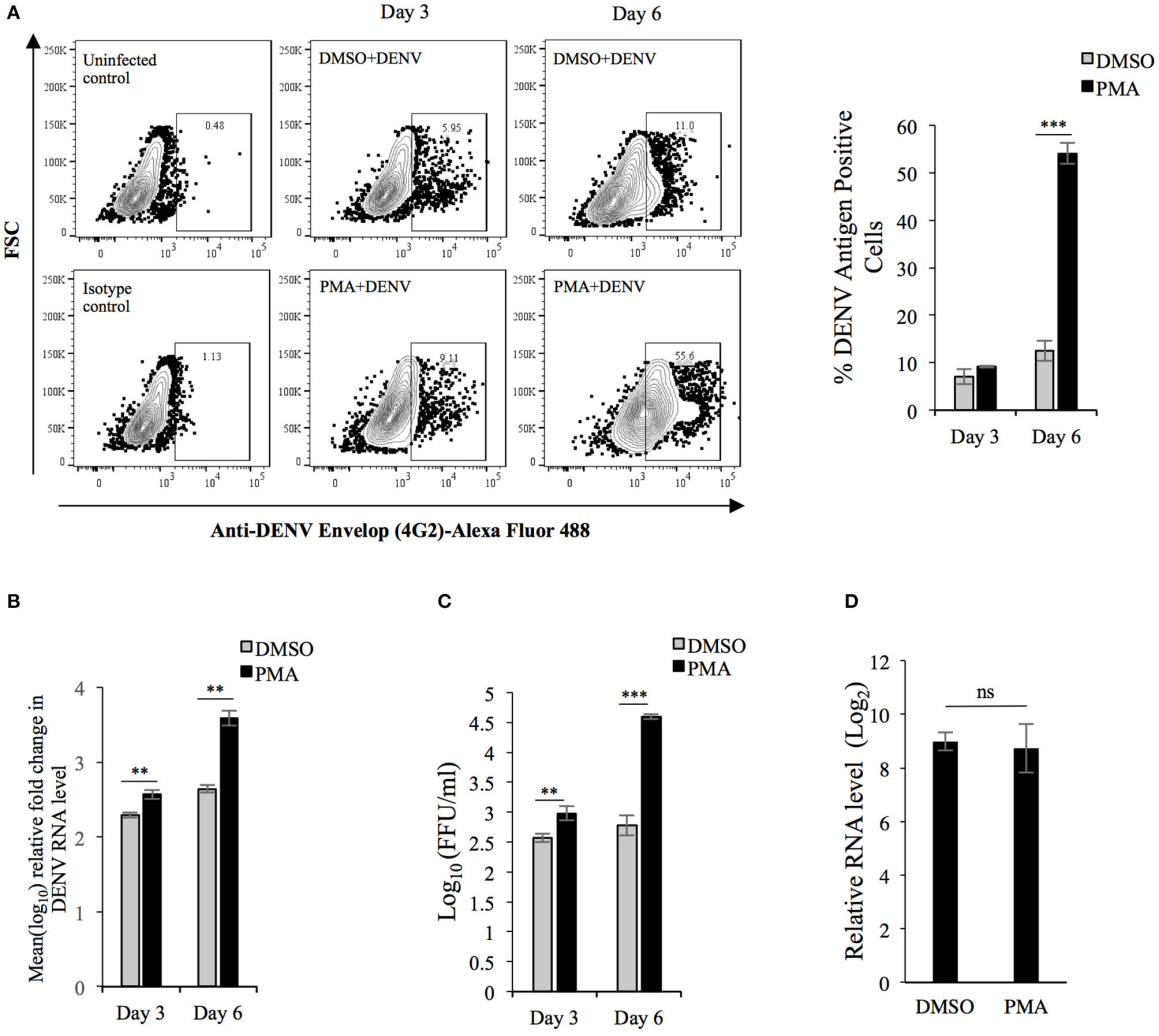
DENV replication is higher in K562 cells undergoing PMA-induced differentiation: **(A)** K562 cells infected with DENV at an MOI of 0.1 were maintained in growth media supplemented with either DMSO or 50 nM PMA and fixed with PFA at 0 h or 3 or 6 days post-infection. The cells were then immune-stained for intracellular DENV antigen and the fluorescence quantified by flow cytometry. The fluorescence in cells fixed at 0 h was used to gate for DENV antigen positive cells. The number of antigen positive cells were quantified and plotted. **(B)** Total RNA from K562 cells treated similarly as in panel A was used for real-time PCR comparison of DENV RNA level at 3 or 6 days to that at 0 h, normalized to that of GAPDH mRNA. The value at 0 h was taken arbitrarily as 1 and that at other time points expressed as fold-change (FC) over that. **(C)** The culture supernatant of cell infected with MOI of 0.1 and subsequently treated as in **(A)** was collected and used for quantification of focus-forming unit/ml (FFU/ml) on Vero cell monolayers. **(D)** Uninfected K562 cells treated with either DMSO or PMA for 3 days were washed with PBS and incubated in either mock- or DENV inoculum (10 MOI) on ice for 2 h. The cells were then transferred to 37°C and further incubated for 2 h. Subsequently cells were washed, treated with 0.25% trypsin for 5 min and the total RNA extracted and used in real-time PCR for comparison of DENV genomic RNA normalized to GAPDH transcripts. The level in mock-infected cells was taken as 1 and that in DENV infected cells calculated as fold-change over that. For Panels A to D, the error bars represent standard deviation obtained from 3 independent experiments. The significance was calculated by Student's *t*-test (**, ***, respectively, indicate *P*-values <0.01 and <0.001 and ns, not significant).

In order to investigate how the PMA-induced changes in K562 cells might affect DENV replication, K562 cells were infected with the virus and immediately transferred to culture media supplemented with either PMA or DMSO (vehicle). The level of viral antigen accumulated after 3- or 6-days post-infection (p.i.) was compared to that at 0-h p.i. as the index of replication. The result showed that although the viral protein accumulated at 3 days p.i was comparable between PMA-treated and DMSO-treated cells, a significant difference between them was observed at day 6 (Figure 2A). In order to check if this correlated with higher degree of genomic RNA replication, the level of viral RNA was compared by real-time PCR while the infectious virus released into culture supernatant was quantified by Focus-forming unit (Ffu) assay. A significantly higher degree of replication and virus release was observed in PMA-treated cells compared to control (Figures 2B,C). Flavivirus maturation is dependent on cleavage of the structural protein prM protein by the host protease Furin, activity of which modulates the infectivity of virion particles (Elshuber et al., 2003). To rule out the possibility that higher infectious titer is because of more virion particles rather than higher infectivity of the same number of virion particles, the viral RNA in culture supernatant was compared by real-time PCR. As expected, the comparison showed more virion particles produced from PMA-treated cells compared to control (Supplementary Figure 1). Since higher replication in PMA-treated cells can be due to either a more conducive milieu for replication or higher rate of entry during secondary infection, the rate of viral entry in cells undergoing differentiation was compared to that in undifferentiated cells. The result showed a comparable entry into cells irrespective of the nature of earlier pharmacological treatment, implying that higher replication in PMA-treated cells occurs through a post-entry mechanism (Figure 2D). Intriguingly, PMA activates Protein kinase C (PKC) which is known to repress Dengue replication by phosphorylation of the viral NS5 protein (Noppakunmongkolchai et al., 2016). Further, no promotion of viral replication was observed by sodium butyrate-treatment of K562 cells (Supplementary Figure 2). All of these results suggest differentiation of K562 cells into MKs generates an intracellular milieu, which is more conducing for viral replication.

### DENV Infection Interferes With Transcriptome Alterations That Accompany Differentiation

**Figure 3:**
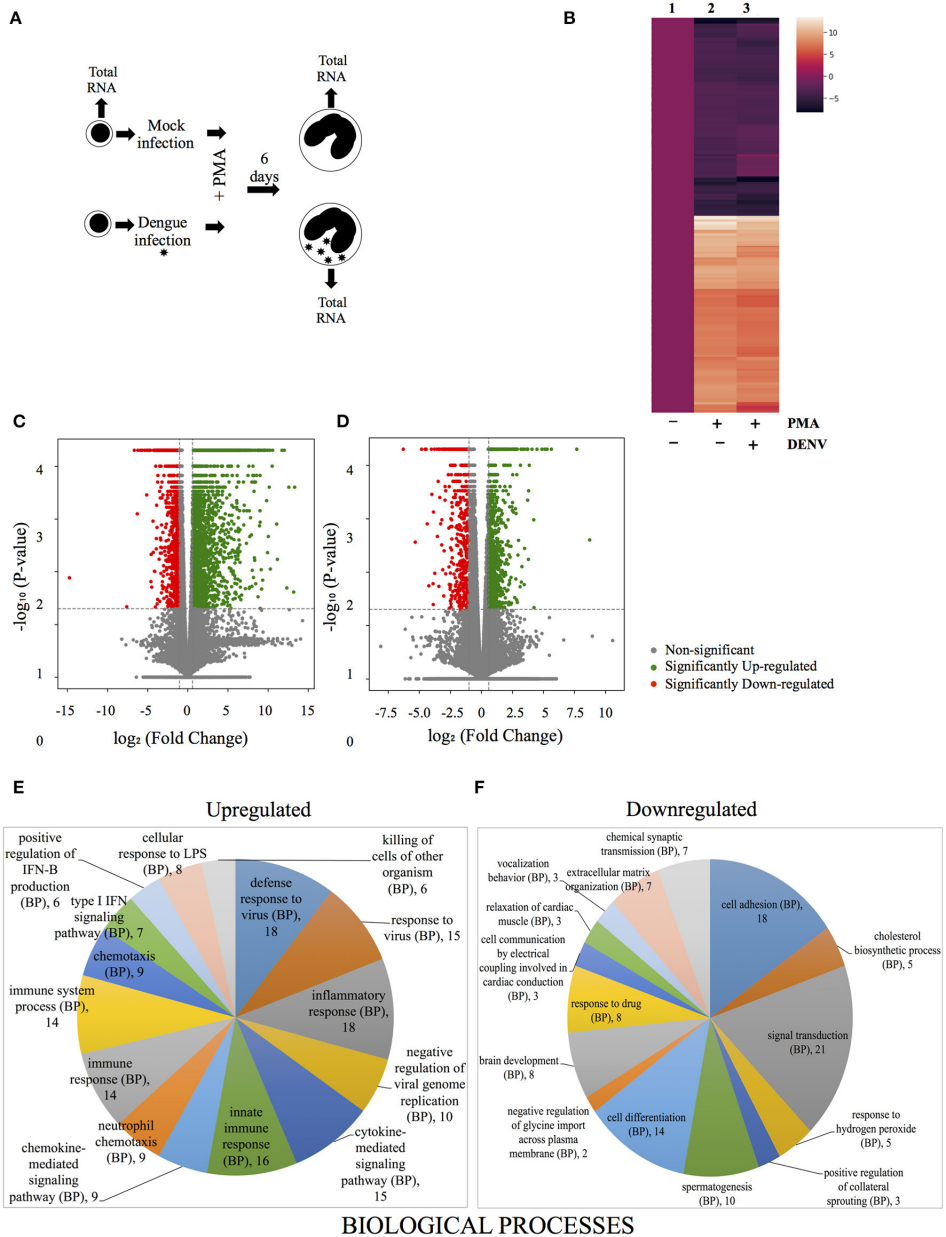
Comparison of transcriptome between uninfected and DENV-infected differentiating cells. **(A)** Schematic representation of the steps for analysis of differential gene regulation in uninfected or DENV-infected K562 cells, undergoing PMA-induced differentiation compared to uninfected cells at 0 h. **(B)** Heatmap depiction of the extent of differential regulation in the top 200 genes deregulated by PMA in uninfected cells (compare column 2 and 1) or in DENV-infected cells (compare columns 3 and 1). The transcript level at 0 h is represented by column 1 while that after 6 days of PMA treatment of uninfected or DENV-infected cells is represented by column 2 or 3, respectively. The fold change in the transcript level were transformed to the logarithmic value to the base of 2 and plotted. **(C,D)** Volcano plots of differentially regulated genes. **(C)** All genes differentially regulated in uninfected cells after 6 days of PMA-treatment compared to those at 0 h. **(D)** All genes that show differential regulation between uninfected and DENV-infected cells after 6 days of PMA-treatment. For both panels the differentially regulated genes were plotted with their corresponding Log_2_ (fold-change) in the X-axis and the -Log_10_ (*P*-value) of deregulation in the Y-axis. The vertical and horizontal broken lines, respectively, depicts the cut off-value in extent of Log_2_ (fold-change) to be either ≥0.58 or ≤ −1 and -Log_10_ (*P-*value) of ≥1.3 (*P*-value of ≤ 0.05), for genes to be considered significantly deregulated. The significantly upregulated and downregulated genes are marked with green and red circles, respectively, while genes that do not show significant deregulation by both criterion are marked with gray circles. **(E,F)** The differentially regulated genes shown in **(D)** were classified according to their enrichment, based on Gene Ontology (GO) term of “Biological process,” using GeneCodis (Nogales-Cadenas et al., 2009). The top 10 enriched GO terms were represented by pie charts with the size of the slices being numerically proportional to the number of genes under each term. The upregulated and downregulated genes are represented in different pie-charts as indicated. The legend indicating each slice shows the GO term and the number of genes classified under it.

Megakaryopoiesis involves dramatic changes in gene expression, which may be responsible for creating a better milieu for virus replication. To investigate this, total RNA was isolated from either mock-infected or virus infected cells differentiating in the presence of PMA for 6 days. Similarly total RNA was isolated from uninfected K562 cells before addition of PMA and these were treated as 0 h samples (Figure 3A). The transcriptome in these total RNA was characterized by Next-generation sequencing, as described in materials and methods. A comparison of the transcriptome between uninfected cells differentiated for 6 days with that of cells at 0 h showed differential expression in a total of 49,272 genes and a comparison of the top 100 deregulated genes (either upregulated or downregulated) between uninfected and DENV-infected cells differentiated for 6 days showed lack of any dramatic difference (Figure 3B). We then proceeded to set a threshold of expression changes and thereby, only transcripts, expression of which were either upregulated by ≥1.5 fold or downregulated to ≤ 0.5 of their level in control (0 h), with a *P*-value of at least 0.05, were considered significantly changed. Using such a criteria PMA was observed to induce differential regulation in 5,714 genes (Supplementary Table 1). A volcano plot shows the distribution of genes which were differentially regulated by PMA after 6 days of differentiation (Figure 3C). Next we compared the significantly regulated genes between uninfected and DENV-infected cells differentiated in the presence of PMA for 6 days, to see the transcript changes induced by virus infection (Figure 3D). Pathway enrichment analysis was performed to segregate the dysregulated genes based on biological processes, cellular components and molecular functions (Figures 3E,F; Supplementary Figures 3A,B). The genes which were observed to be differentially regulated between the uninfected and infected cells were analyzed by GeneCodis with respect to the biological process in which they are involved (Figure 3E). As would be expected the analysis showed innate antiviral and inflammatory genes to be upregulated (Figure 3E; Supplementary Table 2)

On the other hand, genes involved in cell differentiation, cell adhesion and response to hydrogen peroxide were downregulated in the infected cells (Figure 3F). When the genes upregulated by Dengue infection in differentiated cells was examined by Chromatin Immuno-precipitation (ChIP-X) enrichment analysis using the Harmonizome database many targets of the transcription factor NFE2L2, including MYO16, DSC3, CPT1A, MAGI1, TMEM14A, AK5 were observed (Supplementary Figure 4) (Lachmann et al., 2010; Ma, 2013; Rouillard et al., 2016). In addition, a real-time PCR based comparison in the level of other bona-fide targets of NFE2L2, suggested this transcription factor to be activated in differentiated cells that were infected compared to uninfected ones, although no change in the expression of NFE2L2 transcript was observed (Supplementary Figures 5, 6). Since NFE2L2 activated genes is known to suppress ROS levels this indicated that DENV infection might be interfering with PMA-induced accumulation of this critical messenger in MKs.

### Suppression of ROS Accumulation Leads to Increased DENV Replication in Differentiating K562 Cells

**Figure 4:**
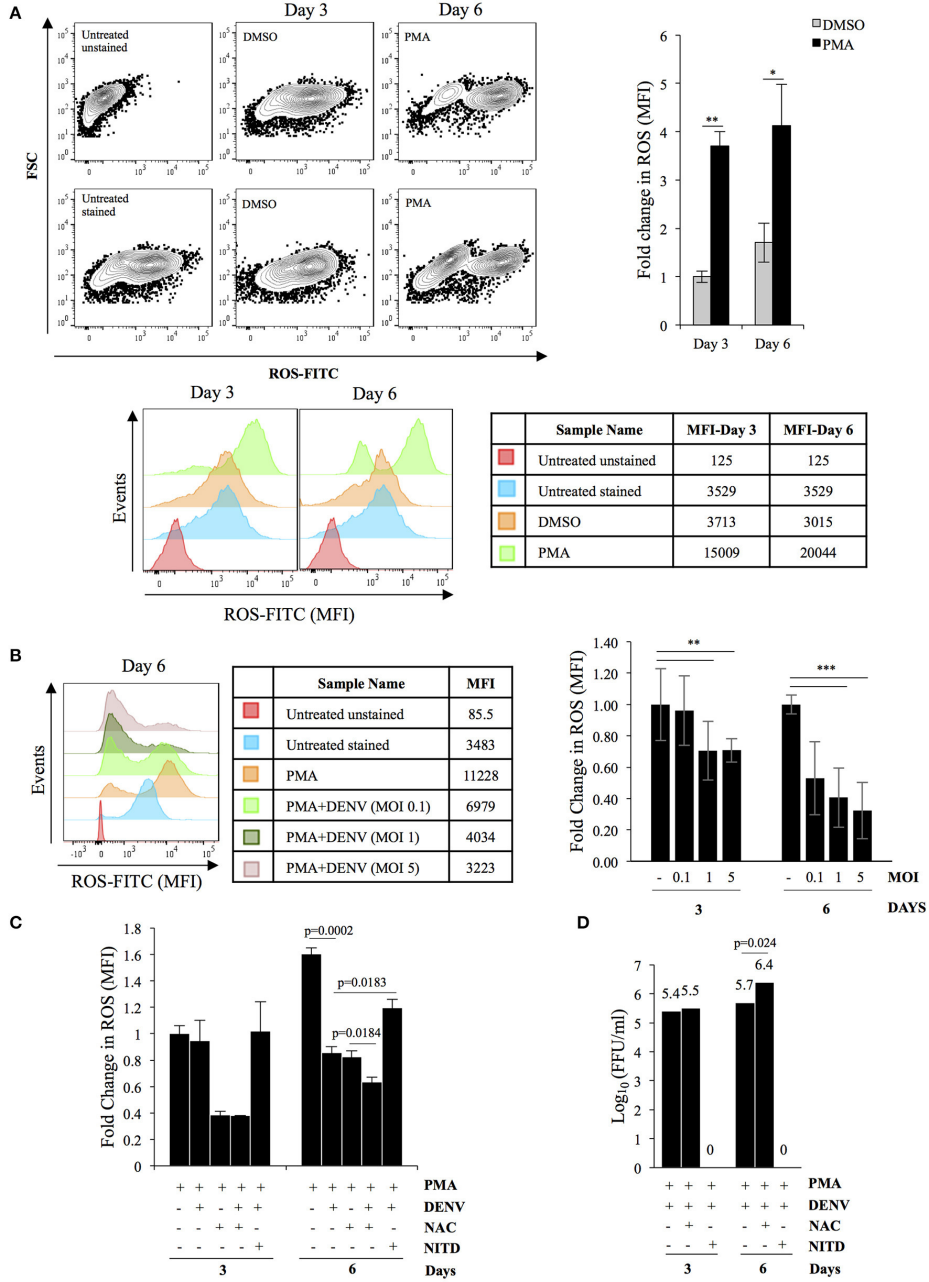
DENV replication suppresses PMA-induced ROS accumulation. **(A)** PMA-induced differentiation leads to ROS accumulation. K562 cells either untreated or treated with either DMSO or 50 nM PMA for a period of either 3 or 6 days, were stained with H_2_DCFDA and the fluorescence quantified in a flow cytometer. The relative fold change in MFI in cells treated with either DMSO or PMA- was plotted. **(B)** DENV infection suppresses ROS accumulation. Cytosolic ROS in K562 cells either uninfected or infected with DENV at the indicated MOI and subsequently treated with PMA for either 3 or 6 days, was quantified by H2DCFDA staining as described above. The ROS level in uninfected cells was arbitrarily taken as 1 and that in others expressed as fold-change with respect to that. **(C)** K562 cells either uninfected or suppression of ROS accumulation is a consequence of virus replication. infected with DENV with an MOI of 1.0 were treated with PMA in the presence of either 3 mM of N-acetyl cysteine (NAC) or 10 μM NITD008 (NITD). At either 3 or 6 days post-infection, the cytosolic ROS quantified by H_2_DCFDA staining as described above. The ROS level in uninfected cells at day 3 was arbitrarily taken as 1 and that in others expressed as fold-change with respect to that. **(D)** K562 cells infected with DENV at an MOI of 1.0 were differentiated with PMA in the presence of either 3 mM of N-acetyl cysteine (NAC) or 10 μM NITD008 (NITD). The supernatant was collected at 3 or 6 days post-infection and the infectious titer of secreted virus quantified by Focus-forming unit (FFU) assay. The Log_10_ of the FFU/ml was calculated and plotted. For Panels A to D, the error bars represent standard deviation obtained from 3 independent experiments and the significance calculated by Student's *t*-test either mentioned or indicated by as * (*, **, and ***, respectively, indicate *P*-values <0.05, <0.01, and <0.001).

**Figure 5:**
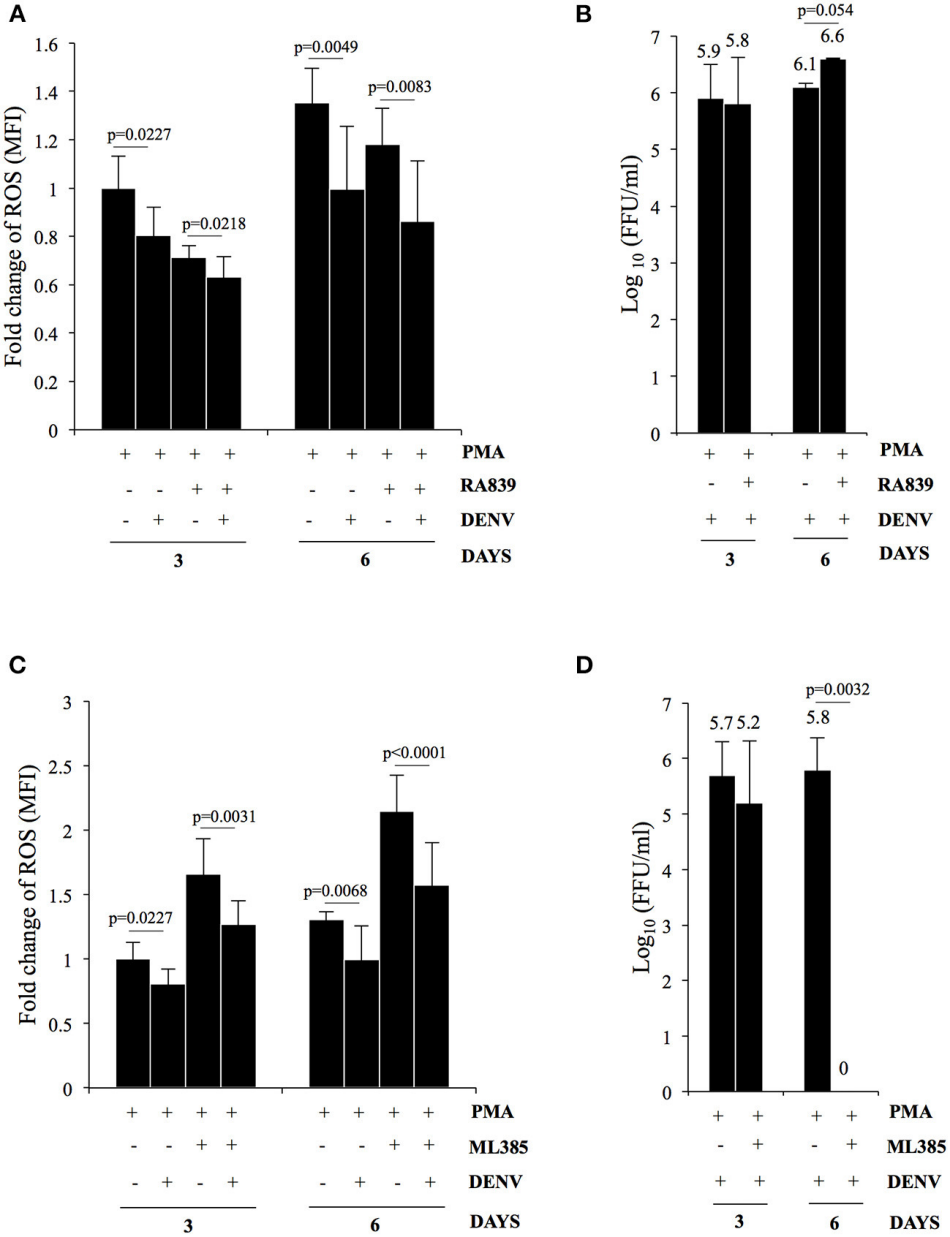
DENV replication suppresses ROS in differentiating cells by upregulation of NFE2L2 activity. **(A)** K562 cells either uninfected or infected with DENV at an MOI of 1.0 were differentiated with 50 nM PMA in the absence or presence of 10 μM of RA839. At 3 or 6 days post-infection the cytosolic ROS was quantified by H_2_DCFDA staining as described above. **(B)** K562 cells infected with DENV at an MOI of 1.0 were differentiated with PMA in the absence or presence of 10 μM of RA839. The supernatant was collected at 3 or 6 days post-infection and the infectious titer of secreted virus quantified by Focus-forming unit (FFU) assay. The Log_10_ of the FFU/ml was calculated and plotted. **(C)** K562 cells either uninfected or infected with DENV at an MOI of 1.0 were differentiated with PMA in the absence or presence of 10 μM of ML385. At 3 or 6 days post-infection the cytosolic ROS was quantified by H_2_DCFDA staining as described above. **(D)** K562 cells infected with DENV at an MOI of 1.0 were differentiated with PMA in the absence or presence of 10 μM of ML385. The culture supernatant was collected at 3 or 6 days post-infection and the infectious titer of secreted virus quantified by Focus-forming unit (FFU) assay. The Log_10_ of the FFU/ml was calculated and plotted. **(A–D)** The error bars represent standard deviation obtained from 3 independent experiments and the *P*-values of significance calculated by Student's *t*-test are indicated.

As reported earlier PMA-induced megakaryopoiesis concomitantly lead to accumulation of intracellular ROS (Figure 4A) (Huang et al., 2014). In an earlier study Olagnier et al. analyzed the transcriptome of DENV infected human monocyte-derived Dendritic cells and observed activation of the NRF2 transcription factor at early time points post-infection (Olagnier et al., 2014). Since DENV infection interfered with deregulation of genes in the biological pathway “Response to hydrogen peroxide,” (PDGFRB, HBA2, HBA1, CRYAB, COL1A1) as observed above, we hypothesized a hyperaccumulation of ROS in infected megakaryocytes compared to uninfected control. To address this, level of ROS accumulation in response to PMA was compared between differentiating cells that were either uninfected or DENV-infected. Surprisingly, the ROS accumulated in infected cells was observed to be lower as compared to uninfected ones, in a MOI dependent manner (Figure 4B). The reduction was found to be similar to that observed in PMA-treated uninfected cells in the presence of N-acetyl cysteine (NAC), a well-characterized quencher of intracellular ROS (Figure 4C). However, this reduction in ROS accumulation was reversed when the infected differentiating cells were treated with NITD008, a known inhibitor of DENV replication, suggesting the suppression of ROS to be a direct fallout of virus replication (Figure 4C). Since ROS can negatively impact virus replication, we were curious to know if this suppression would be beneficial for the virus in the context of our model system (Olagnier et al., 2014). A comparison of the virus secreted into culture supernatant by these cells, in the presence or absence of NAC, showed that super-suppression of ROS accumulation in infected cell by NAC-supplementation moderately increased virus replication (Figure 4D). Since, NFE2L2 activated genes can suppress cellular ROS, we checked the effect of pharmacological compounds that can modulate the function of this TF. Supplementation of RA839, a known activator of NFE2L2, to growth media of PMA-treated cells showed a significant reduction in the ROS accumulation, which was further decreased when the cells were infected with DENV (Figure 5A; Supplementary Figure 7) (Winkel et al., 2015). This suggested that NFE2L2 activation by DENV infection can potentially lead to the suppression in ROS accumulation. On the other hand, supplementation of ML385 a known inhibitor of NFE2L2, increased ROS levels, which was reversed by DENV infection to some extent (Figure 5C; Supplementary Figure 8). In order to address if modulation of NFE2L2 activity and the concomitant regulation of ROS levels influence DENV replication, the titer of virus secreted from these cells was tested by a focus-forming unit (FFU) assay. The result showed that activation of NFE2L2 by RA839 increased virus replication marginally which was not statistically significant (Figure 5B). Conversely, supplementation of NFE2L2 inhibitor ML385 drastically reduced virus replication (Figure 5D). These results indicated that DENV infection in differentiating K562 cells increases the activity of NFE2L2 leading to a reduction in ROS level and increase in virus replication.

### Reduced Expression of MK Specific Surface Markers; CD41/61 Heterodimer and CD 61 in DENV Infected Cells

**Figure 6:**
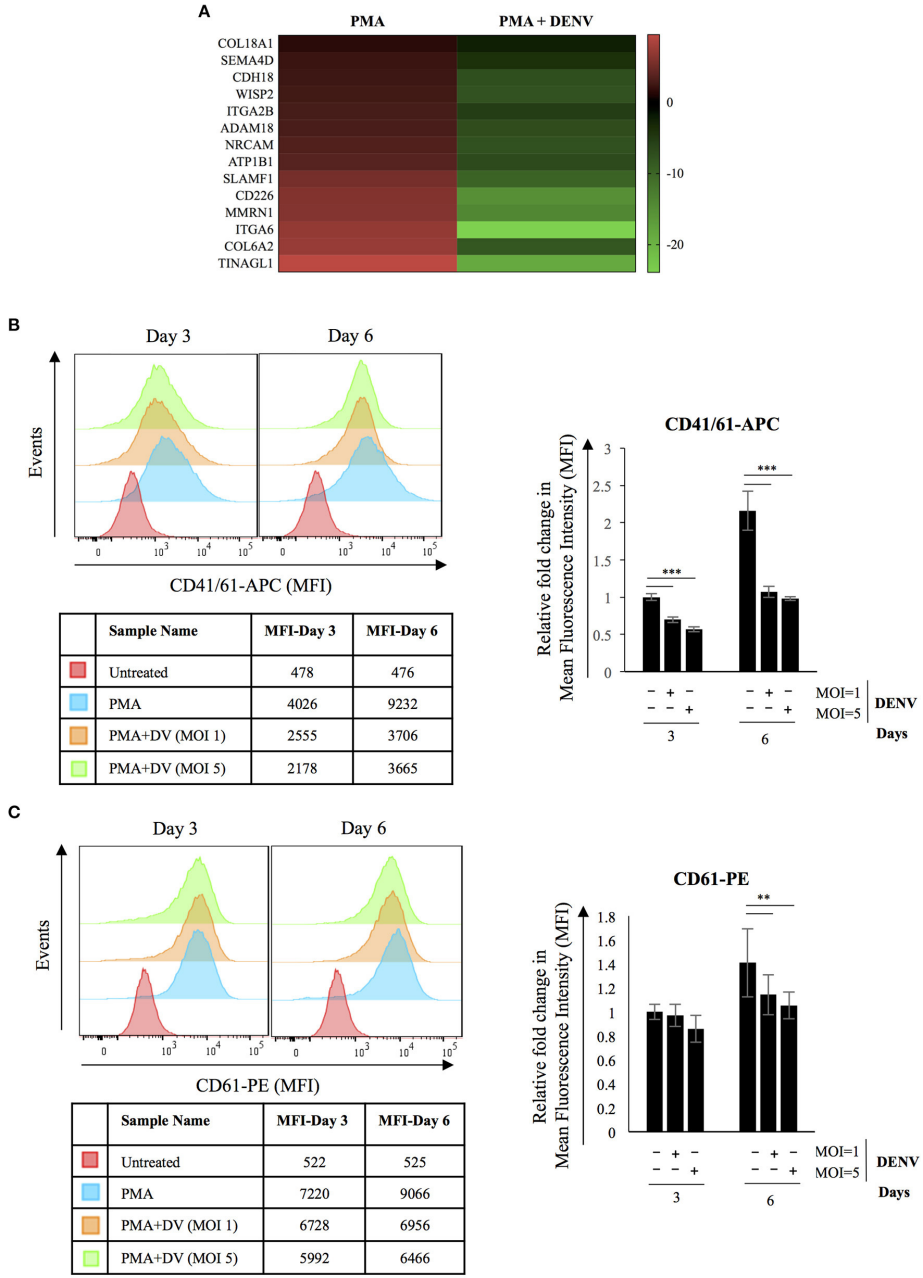
DENV replication reduces expression of MK-specific surface markers in differentiating cells. **(A)** Heatmap showing transcriptional downregulation of genes associated with GO biological process; Cell Adhesion (GO: 0007155) in infected cells differentiated with PMA for 6 days when compared to uninfected controls. **(B,C)** K562 cells either uninfected or infected with DENV at the indicated MOI were differentiated with PMA. At 3 or 6 days post-infection, the surface expression of the indicated marker proteins was quantified by fluor-conjugated primary antibodies in a flow cytometer. The mean fluorescence intensity (MFI) corresponding to each marker was plotted. The error bars represent standard deviation obtained from 3 independent experiments. The significance was calculated by Student's *t*-test (**, ***, respectively, indicate *P*-values <0.01 and <0.001).

Cell adhesion emerged as a major biological process whose genes were dysregulated in DENV infected differentiating cells (Figure 6A). Several studies have documented an essential role of integrins in modulating cell behavior due to their adhesion properties and role in initiating signaling cascades. Therefore, we checked the expression of MK specific markers in infected differentiating cells and the results showed DENV replication to significantly suppress the PMA-induced increase in surface expression of CD41/61 and CD61 (Figures 6B,C). It also indicates that the reduction in ROS level interferes with differentiation by decreasing expression of critical surface markers and cell attachment molecules (Hirose et al., 2013). It would be interesting to study the mechanism of this inhibition so that it can be reversed as a therapeutic approach.

## Discussion

DENV is known to replicate in multiple cell types and bone marrow MKs have been suggested to be particularly permissive. In this article, we report a potential molecular basis of such clinical observations, using a known *in vitro* model of MK differentiation based on human K562 cells. Our results suggest that virus replication is promoted in differentiating MKs, although many innate immune genes are activated as part of the differentiation process. Megakaryopoiesis involves dramatic changes in gene expression and comparison of the transcriptome between differentiating cells that are either uninfected or infected, indicated virus replication to promote the expression of cellular ROS mitigating pathway genes that are under the regulation of the transcription factor NFE2L2. NFE2L2 is a known activator of genes that suppress accumulation of ROS, and as a corroboration of its activation in differentiating cells that were infected showed lower accumulation of ROS when compared to their uninfected counterparts. Further, pharmacological manipulation of ROS level either by a quencher of ROS or activator of NFE2L2 or inhibitor of NFE2L2, showed NFE2L2 mediated ROS suppression in infected cells to be beneficial for virus replication.

*In vitro* models of MK differentiation include human cell lines like HEL, Meg01 and K562, among which HEL and K562 represent Megakaryocyte-Erythrocyte progenitors. In K562, differentiation into either lineage starts with a cease in cell division although subsequent cellular and molecular changes are distinct. Earlier reports suggest PMA to influence replication in a positive or negative manner depending on the virus (Polyak et al., 1991; Golding et al., 1994; Lutteke et al., 2010; Seamone et al., 2010; Outinen et al., 2016; Wang et al., 2017). Here, we observed PMA to promote DENV replication by an unknown post-entry mechanism. Incidentally, replication of Parvovirus B19 has been shown to be higher in differentiating erythroblasts (Outinen et al., 2016). Since differentiation involves increased protein synthesis, it is possible that the DENV genomic RNA, which other than lacking a poly-A tail resembles a host cell mRNA, is translated to a higher extent alongside host mRNAs (Tomer et al., 1988; Daffis et al., 2010). ROS accumulation is known to suppress cellular protein synthesis (Ghosh and Shcherbik, 2020). Here we observed a suppression of ROS in infected cells compared to uninfected counterparts. Therefore, suppression of ROS levels through an interaction of the PMA-stimulated pathway and infection-associated host cell response, might be responsible for increased protein synthesis and consequently for higher viral replication.

Contrary to our expectation, differentiation associated transcriptome changes did not lead to suppression of innate antiviral genes but rather their activation (Henke et al., 2008; Wang and Zhang, 2017). Accumulation of intracellular ROS is known to be essential for differentiation of Megakaryocytes (Huang et al., 2014). Intracellular ROS plays crucial role in regulation of protein tyrosine kinases and phosphatases through post-translational modification (Lee et al., 1998; Salmeen et al., 2003). It can also activate multiple cellular pathways including MAPK, NFκB, cell cycle (Zhang et al., 2016). Intracellular ROS can either promote apoptosis by inducing damage to the mitochondrial membrane or inhibit it through oxidation of catalytic site cysteines on executioner proteases like Caspase-3 (Choi et al., 2010). DENV replication has been shown to induce generation of reactive oxygen species in infected cells (Olagnier et al., 2014). ROS is also known to accumulate in host cells following virus infection and it influences viral replication either positively or negatively (Ramezani et al., 2018). Therefore, it was surprising to observe DENV replication suppressing the accumulation of ROS by activating expression of NFE2L2 target genes. However, supplementation of RA839 which would activate NFE2L2 even further did not show any further increase in virus replication, although ML385 the inhibitor of this TF showed a dramatic effect by totally suppressing virus replication. A recent article by Khan et al. showed accumulation of intracellular ROS to negatively correlate with DENV replication (Khan et al., 2021). DENV like some other flaviviruses, is known to induce accumulation of unfolded/misfolded proteins in the ER lumen leading to the activation of three ER-membrane associated sensors, namely PKR-like ER kinase (PERK), Inositol-requiring enzyme 1 (IRE1a) and Activating transcription factor 6 (ATF6). These sensors then initiate unique pathways and activate genes to mitigate the ER-stress and this response is termed as Unfolded protein response (UPR) (Lee et al., 2018). However, we did not observe any transcriptional signature of UPR, either during differentiation of uninfected cells or by a comparison of differentiating cells that were either uninfected or infected (data not shown). Although stimulation of PERK is known to activate NFE2L2, it is not clear if such a mechanism is responsible here (Cullinan and Diehl, 2004).

The platelet membrane is derived from that of the MK mother cells (Machlus et al., 2013). Therefore, a potential suppression of platelet specific surface markers in DENV-infected MKs suggest either attenuation of biogenesis or formation of platelets with insufficient expression of these genes which might have a repercussion on the hemostatic function of these platelets (Perutelli and Mori, 1997). In addition to that, suppression of the level of transcripts which are preferentially relocated from the mother cells to anucleate platelets, indicate formation of defective platelets (Rowley et al., 2012). It would therefore be interesting to extend this study by studying the transcriptome in platelets from Dengue virus patients so that level of different Megakaryocyte derived transcripts can be compared with healthy subjects.

## Conclusion

Our study shows a potential mechanism by which Megakaryocytes serve as a site for massive Dengue virus replication that contributes to high viremia and attenuated platelet biogenesis. Replication by multiple viruses in their host cell leads to accumulation of intracellular reactive oxygen species (ROS), which often results in activation of apoptosis. Accumulation of ROS positively correlates with Megakaryocyte differentiation. DENV replication in differentiating cells interferes with multiple cellular and molecular changes that are associated with differentiation. We observed a reduction in ROS in infected and differentiating cells through an apparent activation of NFE2L2 transcription factor activity. Further, this reduction in ROS promoted virus replication while simultaneously interfering with optimum differentiation of the megakaryocyte mother cell.

## Materials and Methods

### Cell Culture, Drugs, and Virus

K562 cells were procured from the American Type Culture Collection (ATCC) and cultured at 37°C, 5% CO_2_ in Iscove's modified Dulbecco's media (IMDM) supplemented with Penicillin (100 U/ml), Streptomycin (0.1 mg/ml) and 10%-Fetal Bovine Serum (FBS). The cell line was authenticated using PCR-based species identification and STR profiling. Vero and C6/36 cells were procured from the cell line repository of the National Center for Cell Sciences (NCCS), India and cultured, respectively, in Minimum Essential Medium (MEM) and L15 cell culture medium supplemented with 10% FBS. C6/36 cells were maintained at conditions of 28°C and atmospheric CO_2_. All cells were regularly checked for visible bacterial and fungal contamination or Mycoplasma contamination by PCR-based detection from cell-free culture supernatant.

Dengue virus serotype 2 (strain NGC) was amplified in C6/36 and the culture supernatant was used as inoculum. The infectious titer of the virus was determined by Focus-forming unit (FFU) assay in Vero cells. For all infections, the inoculum was diluted in the respective culture media supplemented with 2% FBS, and the cells were incubated with the inoculum for 2 h at the respective culture conditions of temperature and CO_2_ concentration, with intermittent rocking.

Phorbol-12 Myristate-13 acetate (PMA, Sigma Aldrich), N-Acetyl Cysteine (NAC, Sigma Aldrich), NITD008 (Sigma Aldrich), RA839 (Tocris), ML385 and Sodium Butyrate (Sigma Aldrich) were diluted in recommended vehicles and stored as single use aliquots at −20°C. Fresh dilutions were used for every experiment.

50 nM of PMA was added to the culture medium to promote the differentiation of K562 cells towards megakaryopoiesis.

### Focus-Forming Unit Assay

Vero cell monolayers in 24-well plate were infected with 10-fold serial dilutions of virus inoculum. The inoculum was incubated with cells for 2 h at 37°C and 5% CO_2_ with intermittent rocking. The inoculum was then discarded and complete MEM added to each well followed by incubation for 48 h at 37°C and 5% CO_2_. Subsequently the cells were washed with phosphate-buffered saline (PBS), fixed with 2% para-formaldehyde (PFA) and permeabilized with PBS supplemented with 0.1% Triton-X-100 and 1% bovine serum albumin (BSA). The permeabilized cells were washed with PBS and sequentially incubated anti-DENV primary antibody (dilution 1:400 of Mab8705, Millipore) and anti-Mouse Alexa-488 conjugated secondary antibody (dilution 1:500; ThermoFisher Scientific) both diluted in PBS supplemented with 1% BSA. After incubation with primary antibody, the cells were washed twice with PBS before addition of secondary antibody dilutions. The fluorescent foci were visualized and manually counted using a fluorescence microscope and the titer calculated as FFU/ml.

### Flow Cytometry Analysis

All flow cytometry analysis was performed in a BD FACS Canto II flow cytometer (BD Biosciences) under standard conditions and all raw data analyzed using Flow-Jo software. For immune staining of intracellular DENV antigen, K562 cells were washed with ice-cold PBS before fixation with PBS supplemented with 2% PFA and permeabilization with the same buffer containing 0.1% Triton-X-100. The permeabilized cells were sequentially stained with dilution of 4G2 (purified IgG2a mAb produced in the lab from Hybridoma-HB112) antibody and anti-mouse Alexa-488 conjugated secondary antibody. For immune staining of surface markers, cells were washed with PBS and incubated with dilutions of fluor-conjugated primary antibodies, specific to either human CD61-Phycoerythrin (PE) (BioLegend) or CD41/61-(Allophycocyanin) APC (BioLegend). Antibodies were diluted in staining buffer (PBS with 1%BSA and 0.02% Sodium Azide) and cells were incubated for 1 h at 4°C. Subsequently, the cells were washed with PBS and analyzed by flow cytometry.

Propidium iodide staining for polyploidy analysis: Cells were harvested by centrifugation, washed twice with ice-cold phosphate-buffered saline (PBS) and fixed with 70% ethanol at 4°C for 30 min. After a PBS wash cells were treated with 100 μl PBS supplemented with RNase-A (200 μg/ml) and incubated at 37°C for 30 min. Cells were then washed using PBS and the genomic DNA stained with Propidium iodide (50 μg/ml).

### RNA Extraction and Quantitative RT-PCR

Total RNA from 1 × 10^6^ K562 cells were extracted using Trizol (Takara) and purified with Qiagen RNAeasy mini kit (Qiagen), as per manufacturer's instructions. Viral RNA from cell-free culture supernatant was isolated using QIAmp viral RNA mini kit (Qiagen) as per manufacturer's instructions. 1.0 ug of total RNA was reverse-transcribed with ImProm-II reverse-transcriptase (Promega, USA) and random hexamers (Sigma) as per manufacturer's protocol, and the cDNA diluted with nuclease-free water before use for real-time PCR. Real-time PCR was performed with 2 × SYBR mix (Takara) in a QuantStudio-6 Flex Real-Time PCR System (Applied Biosystems) using the default run program.

### Confocal Microscopy

K562 cells were washed with PBS, fixed and permeabilized as described earlier for immunostaining purposes. The cells were stained with Alexa Fluor-568 Phalloidin (Invitrogen, Thermo fisher scientific) at room temperature for 20 min to stain for F-Actin followed by PBS wash. Cells were then mounted onto glass slides using ProLong Gold Antifade Mountant supplemented with DAPI (Invitrogen, Thermo fisher scientific). The fluorescence was observed and imaged in a FLUOVIEW FV3000 confocal microscope (Olympus).

### Measurement of Intracellular Reactive Oxygen Species

The intracellular ROS accumulated was measure using CM-H2DCFDA probe (Life Technologies) as per manufacturer's instructions. Briefly, K562 cells were washed with PBS and then incubated in PBS supplemented with CM-H2DCFDA as per manufacturer's instructions. The cells were then incubated at 37°C, 5% CO_2_ for 30 min and washed twice with PBS. The fluorescence intensity was quantified in a BD FACS Canto II flow cytometer (BD Biosciences) under standard conditions and the raw data analyzed using Flow-Jo software.

### Next-Generation Sequencing

Total cellular RNA was extracted using Trizol and sent to the National Institute of Biomedical Genomics (NIBMG), Kalyani, India for further processing (RNA extraction, rRNA depletion, sequencing library preparation). Illumina HiSeq 2500 System was used to do paired-end sequencing (2 × 100 bp) and reads that met the quality standards (Phred Score <30; FastQC, Version 0.11.9 followed by adapter removal using Trim Galore, Version 0.6.5) were used for further analysis and were mapped to the most recent stable version of the human reference genome GH38 (GRCh38.p5, Ensembl) using Bowtie 2 and Tophat 2.1.1 (Trapnell et al., 2010). Cufflinks 2.2.1 was used to estimate the expression of the assembled transcriptomes (Roberts et al., 2011; Trapnell et al., 2013). For each sample, normalized gene and transcript expression profiles were computed. The FPKM (Fragments Per Kilobases per Million fragments) method was used followed by log_2_ transformation of the value. The gene-level differential expression in different conditions were estimated using the log_2_ transformed FPKM. While identifying differentially expressed genes (DEGs), the uncorrected *p*-value of the test statistic and the FDR-adjusted *p*-value of the test statistic (*q*-value) were also calculated. After Benjamini-Hochberg correction for multiple testing, any gene with a *p*-value higher than the FDR was considered significantly differentially expressed.

## Data Availability Statement

The original contributions presented in the study are publicly available. This data can be found here: https://www.ncbi.nlm.nih.gov/geo/query/acc.cgi?acc=GSE186089.

## Author Contributions

SB has conceptualized, wrote the paper and supervised the study. JK performed the experiments, analyzed the data and edited the manuscript. YR performed the experiments and analyzed the data. VS, NP, and SK helped in analyzing the transcriptome data. DR helped in analysis of flow cytometry data. NK gave critical inputs in editing of the paper. All authors contributed to the article and approved the submitted version.

## Funding

JK was supported by fellowship from the University Grants Commission. YR was supported from fellowship in the Extramural Research Grant (BT/PR22985/MED/29/1168/2016) to SB from the Department of Biotechnology, Govt of India. This work was supported by Extramural Research Grant (EMR/2016/005796) to SB from the Science and Engineering Research Board, Department of Science and Technology, Govt. of India.

## Conflict of Interest

The authors declare that the research was conducted in the absence of any commercial or financial relationships that could be construed as a potential conflict of interest.

## Publisher's Note

All claims expressed in this article are solely those of the authors and do not necessarily represent those of their affiliated organizations, or those of the publisher, the editors and the reviewers. Any product that may be evaluated in this article, or claim that may be made by its manufacturer, is not guaranteed or endorsed by the publisher.

## Supplementary Material

Supplementary Figure 1 K562 cells infected with DENV at an MOI of 0.1 were maintained in growth media supplemented with either DMSO or 50 nM PMA for 3 or 6 days. Subsequently, RNA from supernatant was extracted and purified. The RNA was reverse transcribed with random hexamers and the cDNA used for real-time PCR estimation of DENV RNA. The C_T_ value corresponding to each was normalized to that of 18S rRNA. The normalized value of untreated mock was taken arbitrarily as 1 and those at day 3 and 6 expressed as fold-change with respect to that. The error bars represent standard deviation and the significance was calculated by Student's *t*-test (^*^, ^***^, respectively, indicate *P*-values <0.05 and <0.001).

Supplementary Figure 2 K562 cells infected with DENV at an MOI of 0.1 were maintained in growth media supplemented with either DMSO or 1 mM sodium butyrate and fixed with PFA at 0 h or 3 or 6 days post-infection. The cells were permeabilized and immune-stained for intracellular DENV antigen and the fluorescence quantified by flow cytometry. The fluorescence in cells fixed at 0 h was used to gate for DENV antigen positive cells. The number of antigen positive cells were quantified and plotted. The error bars represent standard deviation and the significance was calculated by Student's *t*-test (^*^ indicate *P*-value <0.05).

Supplementary Figure 3 **(A,B)** Normalized level of gene transcripts between uninfected and infected K562, both of which were differentiated with 50 nM PMA for 6 days, were compared and the genes that were differentially regulated between these sets were tabulated. These genes were analyzed for Gene Ontology (GO) enrichment analysis using GeneCodis to identify the associated Cellular components (CC) and Molecular Functions (MF) terms. The top 10 terms identified in either group are represented as pie charts.

Supplementary Figure 4 Prediction of Transcription Factors regulating the expression of deregulated genes. A heatmap of ChIP-X enrichment analysis performed in the Harmonizome database web interface to query for Transcription Factors (TFs) regulating the genes that were upregulated by ≥1.5 fold with a *q*-value (FDR-adjusted *p*-value) of <0.05, in infected K562 cells differentiated wit 50 nM PMA for 6 days when compared to uninfected controls.

Supplementary Figure 5 Total RNA from K562 cells either uninfected or infected with DENV at the indicated MOI and subsequently differentiated with 50 nM PMA for 3 or 6 days, was extracted and purified. The RNA was reverse transcribed with random hexamers and the cDNA used for real-time PCR estimation of mRNA transcripts from indicated genes. The C_T_ value corresponding to each was normalized to that of GAPDH. The normalized value of untreated mock was taken arbitrarily as 1 and those at day 3 and 6 expressed as fold-change with respect to that. The error bars represent standard deviation and the significance was calculated by Student's *t*-test (^*^, ^**^, respectively, indicate *P*-values <0.05 and <0.01).

Supplementary Figure 6 **(A–F)** Total RNA from K562 cells either uninfected or infected with DENV at the indicated MOI and subsequently differentiated with 50 nM PMA for 3 or 6 days, was extracted and purified. The RNA was reverse transcribed with random hexamers and the cDNA used for real-time PCR estimation of mRNA transcripts from indicated genes. The C_T_ value corresponding to each was normalized to that of GAPDH. The normalized value of untreated mock was taken arbitrarily as 1 and those at day 3 and 6 expressed as fold-change with respect to that. The error bars represent standard deviation and the significance was calculated by Student's *t*-test (^*^, ^**^, respectively, indicate *P*-values <0.05 and <0.01).

Supplementary Figure 7 K562 cells either uninfected or infected with DENV at an MOI of 1.0 were differentiated with 50 nM PMA in the absence or presence of 10 μM of RA839. At 3 or 6 days post-infection the cytosolic ROS was quantified by H_2_DCFDA staining.

Supplementary Figure 8 K562 cells either uninfected or infected with DENV at an MOI of 1.0 were differentiated with PMA in the absence or presence of 10 μM of ML385. At 3 or 6 days post-infection the cytosolic ROS was quantified by H_2_DCFDA staining.

Supplementary Table 1 List of genes that were differentially regulated by PMA-treatment for 6 days in uninfected K562 cells.

Supplementary Table 2 List of genes that were differentially regulated between uninfected and DENV-infected cells both of which have been differentiated by PMA-treatment for 6 days.

Supplementary Table 3 Mean fluorescence intensity values of surface marker expression (CD41/61 and CD61) in K562 cells with or without DENV infection.
