# Supplementary material for: Replication of Dengue Virus in K562-Megakaryocytes Induces Suppression in the Accumulation of Reactive Oxygen Species
Source: Front Microbiol. 2022 Jan 11;12:784070. doi: 10.3389/fmicb.2021.784070 (PMC8787197; doi:10.3389/fmicb.2021.784070)
Supplement: Supplementary Figure 1 — K562 cells infected with DENV at an MOI of 0.1 were maintained in growth media supplemented with either DMSO or 50 nM PMA for 3 or 6 days. Subsequently, RNA from supernatant was extracted and purified. The RNA was reverse transcribed with random hexamers and the cDNA used for real-time PCR estimation of DENV RNA. The CT value corresponding to each was normalized to that of 18S rRNA. The normalized value of untreated mock was taken arbitrarily as 1 and those at day 3 and 6 expressed as fold-change with respect to that. The error bars represent standard deviation and the significance was calculated by Student's t-test (*, ***, respectively, indicate P-values <0.05 and <0.001). [file Presentation_1.PPTX]

## Slide 1
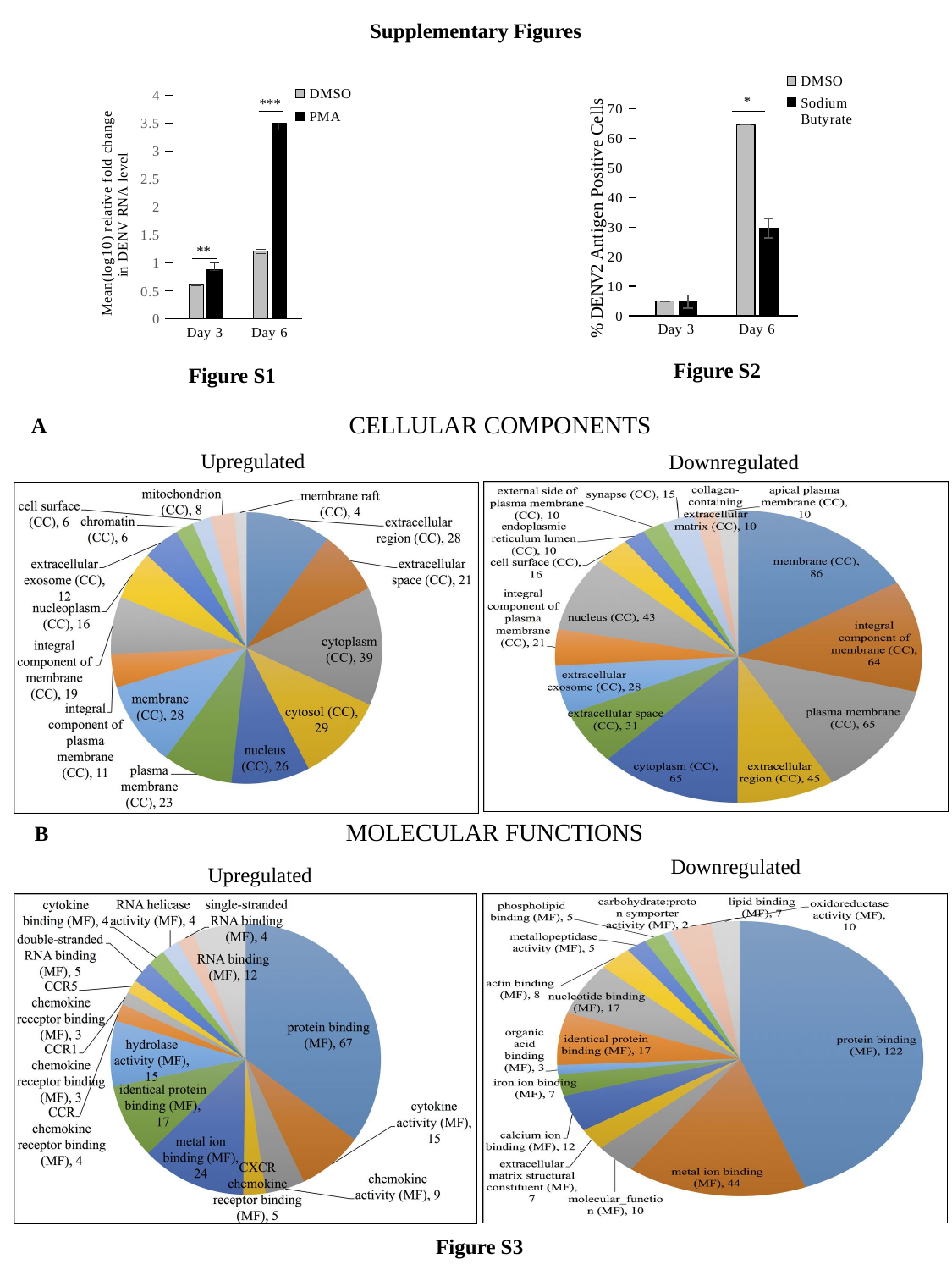

Supplementary Figures
### Chart
| Category | DMSO | Sodium Butyrate |
|---|---|---|
| Day 3 | 4.970000000000001 | 4.819999999999998 |
| Day 6 | 64.65 | 29.65 |
### Chart
| Category | DMSO | PMA |
|---|---|---|
| Day 3 | 0.600209715991649 | 0.883650470351864 |
| Day 6 | 1.208963084193492 | 3.498232617726324 |***
**
Figure S2
Figure S1
CELLULAR COMPONENTS
A
Upregulated
Downregulated
MOLECULAR FUNCTIONS
B
Downregulated
Upregulated
Figure S3

## Slide 2
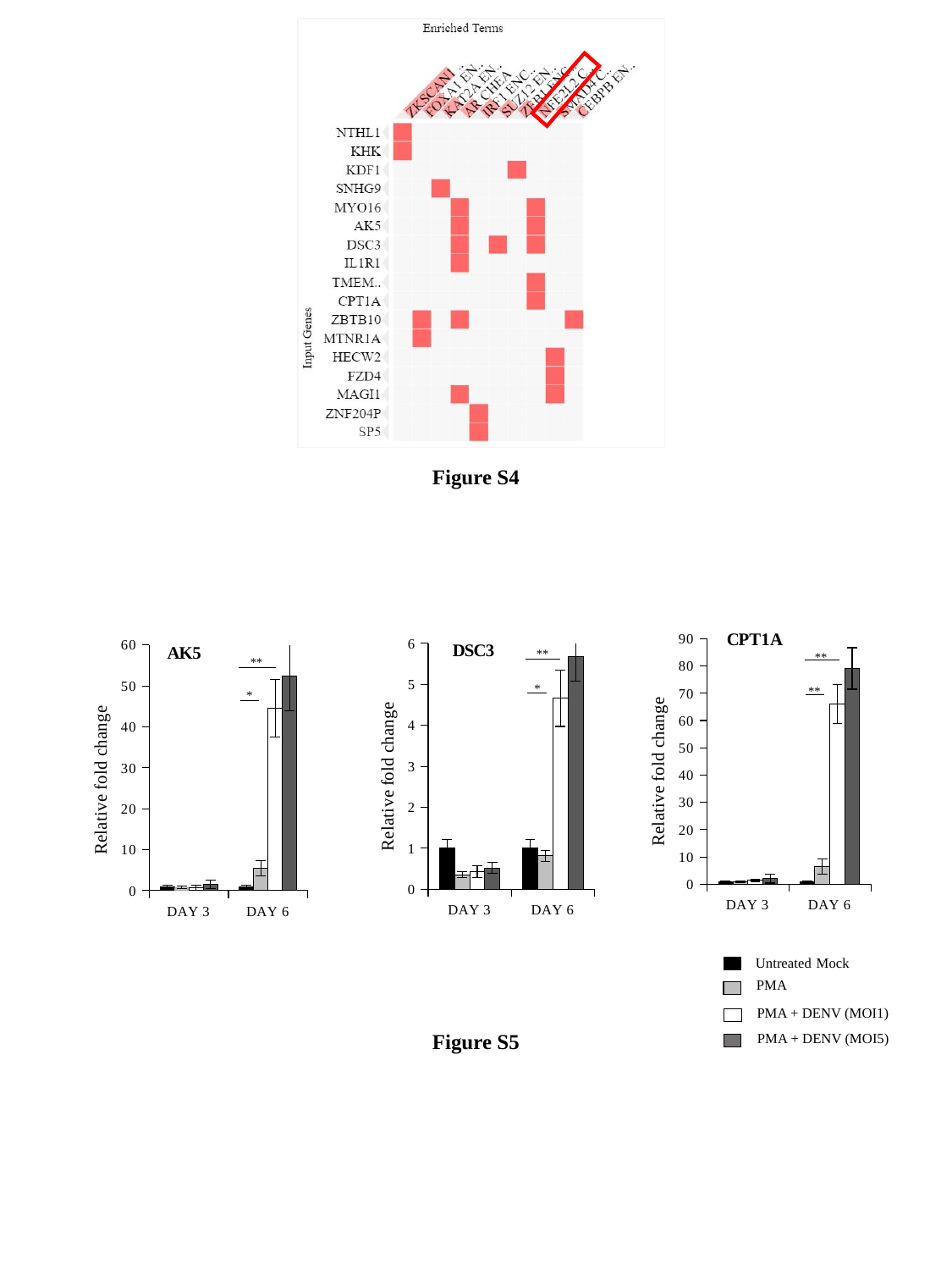

Figure S4
### Chart: CPT1A
| Category | MOCK | PMA | PMA+DV2 MOI 1 | PMA+DV2 MOI 5 |
|---|---|---|---|---|
| DAY 3 | 1.0 | 0.869408345342514 | 1.453417210739736 | 2.11275667792718 |
| DAY 6 | 1.0 | 6.497515918471246 | 66.10764379982157 | 79.05493361614045 |
### Chart: DSC3
| Category | MOCK | PMA | PMA+DV2 MOI 1 | PMA+DV2 MOI 5 |
|---|---|---|---|---|
| DAY 3 | 1.0 | 0.353624038999345 | 0.42699743281703 | 0.521172161936931 |
| DAY 6 | 1.0 | 0.820491219368667 | 4.65982541715119 | 5.67946046421646 |**
### Chart: AK5
| Category | Untreated Mock | PMA | PMA+DENV (MOI 1) | PMA+DENV (MOI 5) |
|---|---|---|---|---|
| DAY 3 | 1.0 | 0.775259280782295 | 0.714540561812749 | 1.586302803564325 |
| DAY 6 | 1.0 | 5.470122664725848 | 44.5342803070354 | 52.362234850039 |*
**
**
*
**
Untreated Mock
PMA
PMA + DENV (MOI1)
PMA + DENV (MOI5)
Figure S5

## Slide 3
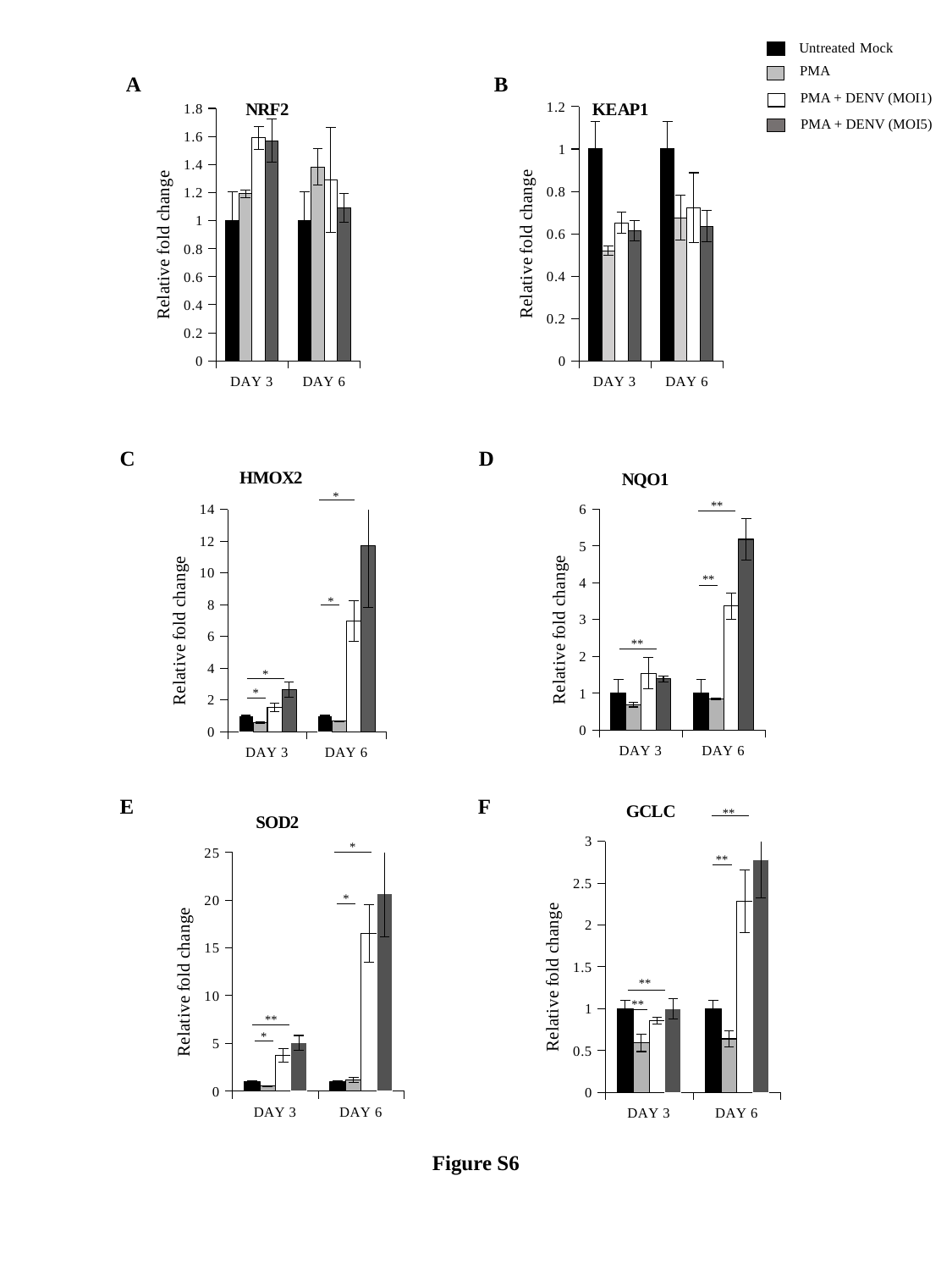

Untreated Mock
PMA
PMA + DENV (MOI1)
PMA + DENV (MOI5)
A
B
### Chart: NRF2
| Category | MOCK | PMA | PMA+DV2 MOI 1 | PMA+DV2 MOI 5 |
|---|---|---|---|---|
| DAY 3 | 1.0 | 1.192012817981618 | 1.589138740908696 | 1.567921850335524 |
| DAY 6 | 1.0 | 1.380848700005606 | 1.292043548695436 | 1.090517628060422 |
### Chart: KEAP1
| Category | MOCK | PMA | PMA+DV2 MOI 1 | PMA+DV2 MOI 5 |
|---|---|---|---|---|
| DAY 3 | 1.0 | 0.520270489712152 | 0.651573382470247 | 0.613872559147587 |
| DAY 6 | 1.0 | 0.675201810732938 | 0.723254473090757 | 0.635705288415332 |C
D
### Chart: NQO1
| Category | Mock | PMA | PMA+DV2 MOI 1 | PMA+DV2 MOI 5 |
|---|---|---|---|---|
| DAY 3 | 1.0 | 0.683101301381505 | 1.534979790160359 | 1.389862203436346 |
| DAY 6 | 1.0 | 0.842162469448455 | 3.366608455546954 | 5.176598646075854 |
### Chart: HMOX2
| Category | Mock | PMA | PMA+DV2 MOI 1 | PMA+DV2 MOI 5 |
|---|---|---|---|---|
| DAY 3 | 1.0 | 0.567193739087466 | 1.509538907002088 | 2.628757821728756 |
| DAY 6 | 1.0 | 0.655923054159788 | 6.955964238233776 | 11.71066628039635 |*
**
**
*
**
*
*
### Chart: GCLC
| Category | Mock | PMA | PMA+DV2 MOI 1 | PMA+DV2 MOI 5 |
|---|---|---|---|---|
| DAY 3 | 1.0 | 0.589677859715819 | 0.856020541249112 | 0.994641034526776 |
| DAY 6 | 1.0 | 0.63850981757776 | 2.2862167205571 | 2.782753009596924 |
### Chart: SOD2
| Category | Mock | PMA | PMA+DV2 MOI 1 | PMA+DV2 MOI 5 |
|---|---|---|---|---|
| DAY 3 | 1.0 | 0.5227419472068 | 3.734680813828107 | 5.03174840917584 |
| DAY 6 | 1.0 | 1.164924157475104 | 16.51376097138276 | 20.66228534891746 |**
*
**
*
**
**
**
*
E
F
Figure S6

## Slide 4
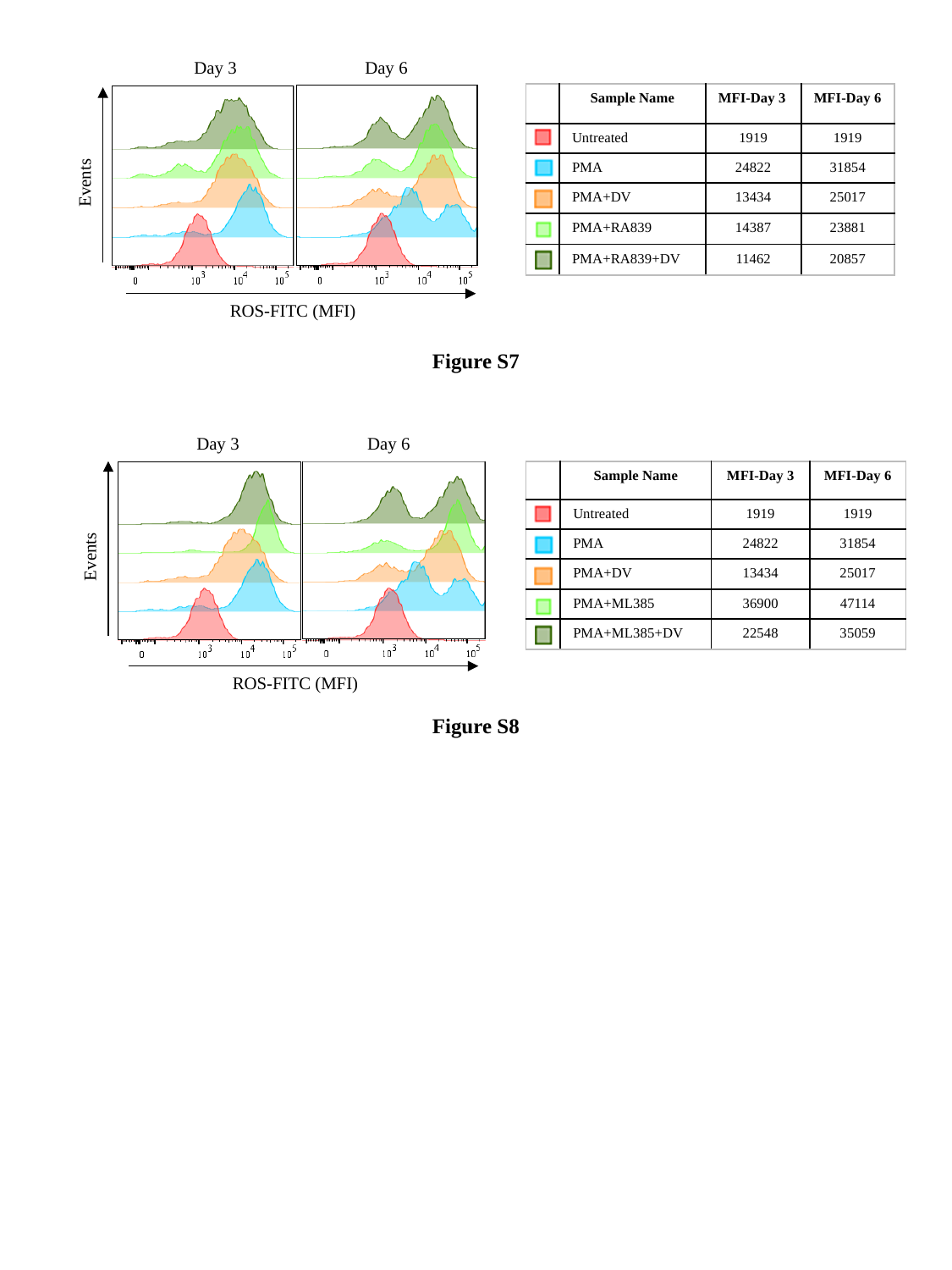

Day 3
Day 6
| | Sample Name | MFI-Day 3 | MFI-Day 6 |
| --- | --- | --- | --- |
| | Untreated | 1919 | 1919 |
| | PMA | 24822 | 31854 |
| | PMA+DV | 13434 | 25017 |
| | PMA+RA839 | 14387 | 23881 |
| | PMA+RA839+DV | 11462 | 20857 |
Events
 ROS-FITC (MFI)
Figure S7
Day 3
Day 6
Events
 ROS-FITC (MFI)
| | Sample Name | MFI-Day 3 | MFI-Day 6 |
| --- | --- | --- | --- |
| | Untreated | 1919 | 1919 |
| | PMA | 24822 | 31854 |
| | PMA+DV | 13434 | 25017 |
| | PMA+ML385 | 36900 | 47114 |
| | PMA+ML385+DV | 22548 | 35059 |
Figure S8
